# Single-cell transcriptomic profiling uncovers key molecular signatures in glioma pathogenesis

**DOI:** 10.3389/fgene.2026.1818742

**Published:** 2026-05-28

**Authors:** Chengcong Gu, Xiaojin Wu, Liang Yang

**Affiliations:** 1 Department of Emergency, Nanhu District People’s Hospital, Jiaxing, Zhejiang, China; 2 Department of Orthopedics, Nanhu District People’s Hospital, Jiaxing, Zhejiang, China

**Keywords:** biomarkers, cellular heterogeneity, glioma, single-cell RNA sequencing, tumor microenvironment

## Abstract

**Background:**

Glioma represents the most prevalent and lethal primary malignant tumor of the central nervous system, characterized by remarkable cellular heterogeneity and poor prognosis. Comprehensive characterization of glioma at single-cell resolution is essential for identifying novel therapeutic targets and improving patient outcomes.

**Methods:**

We performed comprehensive single-cell RNA sequencing (scRNA-seq) analysis on glioma samples obtained from the Gene Expression Omnibus (GEO) database. Advanced computational approaches including principal component analysis (PCA), uniform manifold approximation and projection (UMAP), t-distributed stochastic neighbor embedding (t-SNE), and differential expression analysis were employed to characterize cellular heterogeneity. Gene co-expression network construction and pathway enrichment analysis were conducted to identify functional modules. Candidate biomarkers were validated using quantitative real-time PCR (qRT-PCR) and enzyme-linked immunosorbent assay (ELISA) in GL261 glioma cells, C8-D1A normal glial cells, and intervertebral disc nucleus pulposus cells.

**Results:**

Quality control analysis revealed high-quality single-cell data with median gene counts of 5,437 and UMI counts of 14,207 per cell. A total of 4,753 highly variable genes were identified, and 22 distinct cellular clusters were delineated using the Louvain algorithm. PCA loading analysis identified key contributing genes including cell cycle regulators (CDK1, TOP2A, BIRC5), immune-related genes (C1QA, C1QB, HLA-DRB6), and neural lineage markers (MOBP, MAG, GJB1). Cell type annotation identified seven major populations: astrocytes, oligodendrocytes, microglia, neural stem cells, OPC/immature neurons, pericytes, and T cells. Differential expression analysis uncovered 847 upregulated and 652 downregulated genes (|log_2_FC| > 1, adjusted P < 0.05). Gene co-expression network analysis revealed five major functional modules centered on hub genes including CLEC12A, CLU, AQP4, and S100A16. Pathway enrichment demonstrated significant involvement of cell cycle, Notch signaling, MAPK pathway, and neurogenesis. Experimental validation confirmed that Plp1 was significantly downregulated in GL261 cells (0.38 ± 0.05-fold, P < 0.01), while *F*th1 (2.15 ± 0.28-fold, P < 0.001) and Gm42418 (5.67 ± 0.52-fold, P < 0.001) were markedly upregulated compared to normal glial cells.

**Conclusion:**

This comprehensive single-cell transcriptomic analysis successfully characterized the cellular heterogeneity of glioma, identifying distinct cell populations and molecular signatures. PLP1, FTH1, and GM42418 were validated as potential molecular biomarkers, providing novel insights into glioma pathogenesis and potential therapeutic targets.

## Introduction

Gliomas represent the most prevalent and devastating primary malignant tumors of the central nervous system, accounting for approximately 80% of all malignant brain tumors and exhibiting remarkable cellular heterogeneity, aggressive biological behavior, and dismal prognosis despite multimodal therapeutic interventions (Kinnersley et al., 2018; Melin et al., 2017). The intrinsic complexity of glioma pathogenesis, characterized by profound genetic alterations, dynamic tumor microenvironment interactions, and extensive cellular plasticity, poses significant challenges for both mechanistic understanding and therapeutic development. Despite substantial advances in neurosurgical techniques, radiation therapy, and chemotherapeutic regimens over the past decades, the median survival for patients with glioblastoma, the most aggressive subtype, remains approximately 15 months, underscoring the urgent need for novel diagnostic biomarkers and therapeutic targets (Ostrom et al., 2019).

Recent technological breakthroughs in transcriptomic profiling have revolutionized our understanding of glioma biology at unprecedented resolution. The advent of single-cell RNA sequencing (scRNA-seq) technology has enabled comprehensive characterization of cellular heterogeneity within the tumor microenvironment, revealing previously unrecognized cell subpopulations, developmental hierarchies, and transcriptional programs that drive tumor initiation, progression, and therapeutic resistance (Pombo Antunes et al., 2021; Patel et al., 2014). Single-cell analysis unveils the microscopic cellular diversity and dynamic transcriptional landscapes within individual tumors, enabling the identification of functionally relevant molecular alterations and facilitating the discovery of actionable biomarkers and therapeutic vulnerabilities (Yuan et al., 2023).

The tumor microenvironment of glioma comprises a complex ecosystem of neoplastic cells, immune cells, neural cells, and stromal components that collectively influence tumor behavior and treatment response (Neftel et al., 2019; Johnson et al., 2018). Understanding the cellular composition and molecular characteristics of this microenvironment is crucial for developing effective therapeutic strategies. Previous investigations have characterized glioma at the transcriptomic level, revealing the coexistence of multiple cell states and the dynamic interplay between tumor cells and their microenvironment (Jung et al., 2021; Mackay et al., 2021). However, comprehensive validation across independent platforms—from single-cell transcriptomics to protein-level confirmation—represents a critical step in translating bioinformatic discoveries into clinically relevant biomarkers.

To address these challenges, we conducted a comprehensive single-cell RNA sequencing analysis combined with systematic experimental validation to characterize the cellular complexity of glioma. By leveraging publicly available single-cell transcriptomic data from the Gene Expression Omnibus (GEO) database, we dissected cellular heterogeneity within glioma tissues. Advanced computational analyses, including dimensionality reduction, differential expression profiling, gene co-expression network construction, and pathway enrichment analysis, enabled the identification of candidate molecular biomarkers exhibiting distinct expression patterns across cellular populations. Critically, we validated these bioinformatic findings through quantitative real-time PCR (qRT-PCR) and enzyme-linked immunosorbent assay (ELISA) in established glioma cell lines (GL261) and normal glial cells (C8-D1A), providing robust evidence for the biological relevance of the identified molecular signatures (Alves da Costa and Lang, 2022; Luo et al., 2022).

## Methods

### Single-cell RNA sequencing data processing and quality control

Single-cell RNA sequencing data for this study were obtained from the GEO database (GSM9116755). Initial quality control involved assessing unique feature RNA (nFeature_RNA) and total UMI counts (nCount_RNA) per cell using violin plots. The relationship between sequencing depth and mitochondrial gene percentage was evaluated to identify and remove low-quality cells and potential doublets. Cells with nFeature_RNA below 200 (likely empty droplets) or above 6,000 (likely doublets), as well as cells with mitochondrial gene percentage exceeding 20% were excluded from downstream analyses. The distribution of UMI counts and their relationship with mitochondrial content was visualized using density scatter plots with marginal histograms.

### Identification of highly variable genes

Highly variable genes were identified using the FindVariableFeatures function with the variance-stabilizing transformation (vst) method in Seurat. The top 4,753 genes with the highest standardized variance across cells were selected for downstream analyses. These genes, which capture the most biological variation in the dataset, were visualized by plotting standardized variance against mean expression (log scale), with highly variable genes highlighted in red.

### Dimensionality reduction and cell clustering

Data scaling was performed using the ScaleData function to ensure that highly expressed genes do not dominate downstream analyses. Principal component analysis (PCA) was conducted on the scaled data matrix of highly variable genes, and the first 30 principal components were retained based on elbow plot assessment. PCA visualization with cluster boundaries was generated to reveal initial patterns of cellular heterogeneity. A loading heatmap displaying the top contributing genes to each principal component was constructed to identify major drivers of variance.

Uniform Manifold Approximation and Projection (UMAP) and t-distributed Stochastic Neighbor Embedding (t-SNE) dimensionality reduction were performed using the first 30 principal components to visualize cellular heterogeneity in two-dimensional space. Cell clustering was performed using the Louvain algorithm implemented in the FindClusters function with a resolution parameter of 0.8 (empirically selected after evaluating a range of resolution values from 0.4 to 1.2; the value of 0.8 was chosen based on the elbow plot and the biological coherence of the resulting clusters as assessed by canonical marker gene expression patterns), identifying 22 distinct cellular populations (Clusters 0-21). Three-dimensional PCA visualization was generated to provide an integrated spatial representation of cellular heterogeneity.

Regarding batch effect considerations: as this study analyzed a single sample (GSM9116755), inter-sample batch effects were not a primary concern. Technical biases inherent to single-cell capture—such as cell capture variability and differences in sequencing depth between cells—were mitigated through our quality control pipeline (nFeature_RNA and percent.mt filtering) and by applying variance-stabilizing transformation (VST) normalization in Seurat. Because only one sample was analyzed, no inter-batch correction algorithm (such as Harmony or ComBat-seq) was required or applied. We acknowledge that multi-sample or multi-dataset integration in future studies would necessitate rigorous batch correction to ensure the validity of cross-sample comparisons.

### Cell type identification and differential expression analysis

Cell type identification was performed by examining the expression patterns of canonical marker genes across identified clusters using UMAP feature plots, violin plots, ridge plots, and dot plots. The FindAllMarkers function was employed to identify differentially expressed genes for each cluster compared to all other clusters using the Wilcoxon rank-sum test, with genes showing log_2_ fold-change greater than 0.25 and adjusted P-value less than 0.05 considered as significant cluster markers. Expression patterns of top marker genes were visualized using dot plots and Z-score normalized heatmaps. Cell type composition across clusters was quantified and visualized using stacked bar plots.

### Gene Co-expression network and pathway enrichment analysis

Gene co-expression network analysis was conducted to identify modules of coordinately expressed genes and their hub regulators. Network visualization was performed to illustrate functional relationships within the transcriptional landscape. Differential expression analysis between groups was performed using the FindMarkers function, with results visualized in volcano plots displaying log_2_ fold-change versus -log_10_(adjusted P-value). Pathway enrichment analysis was conducted using clusterProfiler to identify significantly enriched biological pathways, including cell cycle, Notch signaling, MAPK pathway, and neurogenesis. Cell type expression correlation analysis was performed to reveal relationships between different cell populations.

### Cell complexity assessment

Cell complexity was evaluated by analyzing the relationship between gene counts (number of genes) and UMI counts using scatter plots with linear regression. Pearson correlation coefficients were calculated to quantify the relationship. Transcriptional complexity by cluster was assessed by calculating the mean genes detected per cell for each cluster and visualized using horizontal bar plots. The relationship between sequencing depth and gene detection was further examined on a log scale with density coloring to reveal data distribution patterns.

### Cell culture and maintenance

C8-D1A mouse astrocyte cells (ATCC CRL-2541), representing normal glial cells, and GL261 mouse glioma cells (ATCC CRL-2361) were obtained from the American Type Culture Collection. Primary mouse nucleus pulposus cells isolated from intervertebral discs were cultured as representative disc cells. All cell lines were maintained in Dulbecco’s Modified Eagle Medium (DMEM, Gibco) supplemented with 10% fetal bovine serum (FBS, Gibco), 100 U/mL penicillin, and 100 μg/mL streptomycin (Gibco). Cells were cultured in a humidified incubator at 37 °C with 5% CO_2_ atmosphere. Culture medium was changed every 2–3 days, and cells were passaged upon reaching 80%–90% confluence using 0.25% trypsin-EDTA solution. Cells between passages three and eight were used for all experiments.

### Quantitative real-time PCR (qRT-PCR)

Total RNA was extracted from GL261 glioma cells, C8-D1A normal glial cells, and intervertebral disc nucleus pulposus cells using TRIzol reagent (Invitrogen) according to the manufacturer’s instructions. RNA concentration and purity were assessed using a NanoDrop 2000 spectrophotometer. One microgram of total RNA was reverse-transcribed into cDNA using the PrimeScript RT reagent kit (TaKaRa). qRT-PCR was performed on an ABI 7500 Real-Time PCR System using SYBR Green Master Mix (TaKaRa) in a 20 μL reaction volume. The PCR amplification conditions were as follows: initial denaturation at 95 °C for 30 s, followed by 40 cycles of 95 °C for 5 s and 60 °C for 34 s. GAPDH was used as the internal reference gene, and relative expression levels were calculated using the 2^(-ΔΔCt) method. Each sample was analyzed in triplicate. The primer sequences used for RT-qPCR and the validation results of candidate biomarkers are shown in Tables 1, 2, respectively.

**TABLE 1:** Primer sequences for RT-qPCR analysis.

Gene	Forward Primer (5′-3′)	Reverse Primer (5′-3′)
Plp1	TGT​GGC​TCA​GGG​TGA​AAG​AG	CCA​GAG​CAG​CAG​ATA​GCA​CA
*F*th1	TGA​AGA​ACT​TTG​CCC​AGA​TCC	GCC​ACA​TCA​TCT​CGG​TCA​AAG
Gm42418	CTG​ACC​TCA​AGT​GAT​CCA​CCC	AGC​ACA​GTG​GCT​TAG​TTC​CT
GAPDH	AGG​TCG​GTG​TGA​ACG​GAT​TTG	TGT​AGA​CCA​TGT​AGT​TGA​GGT​CA

**TABLE 2:** qRT-PCR validation results of candidate biomarkers.

Gene	C8-D1A (Normal)	GL261 (Glioma)	NP Cells (Disc)
Plp1	1.00 ± 0.08	0.38 ± 0.05**	0.72 ± 0.09
*F*th1	1.00 ± 0.11	2.15 ± 0.28***	1.45 ± 0.18
Gm42418	1.00 ± 0.09	5.67 ± 0.52***	2.13 ± 0.31

Data are presented as mean ± SD (n = 3). **P < 0.01, ***P < 0.001 vs. C8-D1A control cells (one-way ANOVA, with Tukey’s post-hoc test).

### Enzyme-linked immunosorbent assay (ELISA)

Protein expression levels of PLP1, FTH1, and GM42418 in different cell lines were quantified using commercially available ELISA kits according to the manufacturer’s protocols. Briefly, cells were lysed using RIPA buffer containing protease inhibitors, and protein concentrations were determined by BCA assay. Cell lysates (100 μg protein per well) were added to pre-coated 96-well plates and incubated overnight at 4 °C. After washing three times with PBS-T, wells were incubated with specific detection antibodies for 2 h at room temperature, followed by HRP-conjugated secondary antibodies for 1 h. Color development was achieved using TMB substrate, and reactions were stopped with 2M H_2_SO_4_. Absorbance was measured at 450 nm using a microplate reader. All samples were analyzed in triplicate.

### Statistical analysis

All statistical analyses were performed using R software (version 4.2.0) and GraphPad Prism 9.0. Single-cell data analysis was conducted using the Seurat package. Correlation analyses were evaluated using Pearson correlation coefficients. For qRT-PCR and ELISA data, comparisons between groups were performed using one-way ANOVA followed by Tukey’s post-hoc test for multiple comparisons. Data are presented as mean ± standard deviation (SD) from three independent experiments. A two-sided P-value <0.05 was considered statistically significant.

## Results

### Single-cell RNA sequencing quality control and initial clustering

**FIGURE 1:**
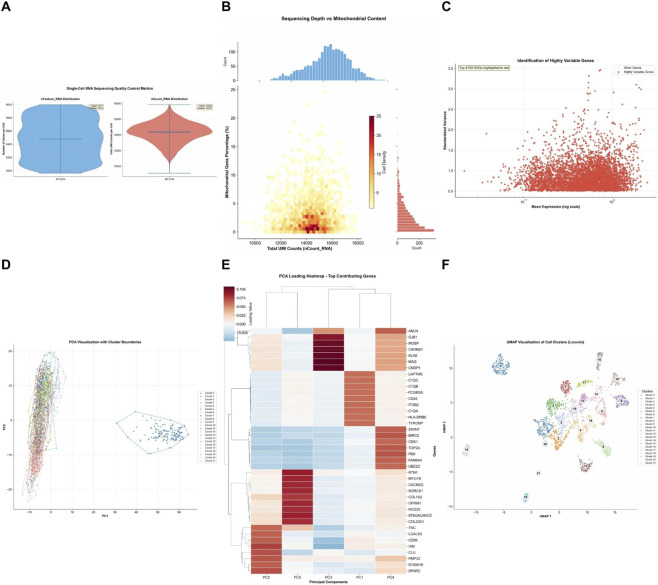
Single-cell RNA sequencing quality control and initial clustering. **(A)** Violin plots displaying the distribution of unique feature RNA (nFeature_RNA) and total UMI counts (nCount_RNA) per cell, showing median values of 5,437 genes and 14,207 UMIs. **(B)** Density scatter plot with marginal histograms showing the relationship between sequencing depth (nCount_RNA) and mitochondrial gene percentage, demonstrating low mitochondrial content indicative of high-quality cells. **(C)** Identification of highly variable genes (n = 4,753) plotted as standardized variance against mean expression on log scale, with highly variable genes highlighted in red. **(D)** PCA visualization with cluster boundaries showing cell distribution in PC1-PC2 space. **(E)** PCA loading heatmap displaying top contributing genes to principal components PC1-PC5, revealing cell cycle regulators, immune markers, and neural lineage genes. **(F)** UMAP visualization of cell clusters identified using the Louvain algorithm, revealing 22 distinct cellular populations (Clusters 0-21).

Initial quality control assessments were performed to ensure data reliability for the glioma single-cell RNA sequencing dataset. Violin plots illustrated the distribution of unique feature RNA (nFeature_RNA) and total UMI counts (nCount_RNA) per cell, revealing a median of approximately 5,437 genes detected per cell and median UMI counts of 14,207, indicating high-quality sequencing depth and robust gene detection (Figure 1A). The relationship between sequencing depth (nCount_RNA) and mitochondrial gene percentage (percent.mt) was evaluated using a density scatter plot with marginal histograms (Figure 1B), demonstrating that the majority of cells exhibited low mitochondrial content (<5%), indicative of healthy cells with minimal contamination or stress-induced artifacts.

Highly variable genes, crucial for identifying cellular heterogeneity, were identified using variance-stabilizing transformation. A total of 4,753 highly variable genes were identified and visualized by plotting standardized variance against mean expression on a log scale, with highly variable genes highlighted in red (Figure 1C). These genes capture the major sources of biological variation in the dataset and were used for subsequent dimensionality reduction analyses.

Principal component analysis (PCA) was performed on the scaled expression matrix of highly variable genes. PCA visualization with cluster boundaries revealed initial patterns of cellular heterogeneity, with cells distributed across multiple distinct groups in PC1-PC2 space (Figure 1D). The PCA loading heatmap displayed the top contributing genes to each of the first five principal components (Figure 1E). Notably, PC2 showed strong contributions from cell cycle-related genes including ZWINT, BIRC5, CDK1, TOP2A, PBK, FAM64A, and UBE2C. PC5 was enriched for immune-related genes such as C1QC, C1QB, FCGR3A, CD53, ITGB2, C1QA, HLA-DRB6, and TYROBP. PC3 and PC1 showed contributions from neural lineage markers including ANLN, GJB1, MOBP, CXCNS1, KLK6, and MAG, as well as structural proteins like VIM, CLU, PMP22, S100A16, and SPARC.

Uniform Manifold Approximation and Projection (UMAP) analysis facilitated cell clustering using the Louvain algorithm, identifying 22 distinct cellular populations (Clusters 0-21) represented by different colored clusters (Figure 1F). The clusters showed clear spatial separation in the UMAP embedding, indicating transcriptionally distinct cell populations with unique molecular signatures.

### Characterization of cell cluster heterogeneity through multi-dimensional analysis

**FIGURE 2:**
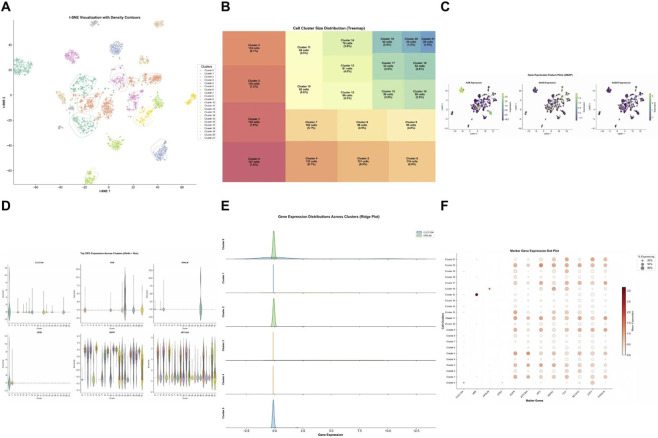
Characterization of cell cluster heterogeneity through multi-dimensional gene expression analysis. **(A)** t-SNE visualization with density contours showing 22 distinct cell clusters with clear spatial separation. **(B)** Treemap displaying cell cluster size distribution, with cluster sizes ranging from 20 cells (Cluster 21, 1.0%) to 188 cells (Cluster 0, 9.4%). **(C)** UMAP feature plots showing expression levels of A2M, AAAS, and AADAF genes across cell clusters. **(D)** Violin plots depicting expression distributions of top differentially expressed genes (CLEC12A, SNB, OPALIN, ODN2, EGFR, AFP1XM1) across clusters. **(E)** Ridge plot showing gene expression distributions of CLEC12A and OPALIN across clusters 0-5. **(F)** Marker gene expression dot plot illustrating percentage of expressing cells (dot size) and average expression level (color intensity) across all clusters.

Further characterization of cellular heterogeneity was performed using complementary dimensionality reduction approaches. T-SNE visualization with density contours confirmed the clustering patterns observed in UMAP, demonstrating robust identification of 22 distinct cell populations with clear spatial separation (Figure 2A). The density contours highlighted regions of high cell density within each cluster, indicating well-defined transcriptional states.

A treemap visualization illustrated the cell cluster size distribution, revealing substantial variation in cluster sizes (Figure 2B). The largest cluster (Cluster 0) contained 188 cells (9.4%), while smaller clusters such as Cluster 21 comprised 20 cells (1.0%). Other major clusters included Cluster 3 (174 cells, 8.7%), Cluster 2 (143 cells, 7.2%), and Cluster 9 (157 cells, 7.8%). This distribution reflects the heterogeneous composition of the glioma tumor microenvironment with distinct cell populations of varying abundance.

Gene expression feature plots on UMAP embedding demonstrated the spatial distribution of specific marker genes including A2M, AAAS, and AADAF across cellular clusters (Figure 2C). These plots revealed cluster-specific expression patterns, with certain genes showing enrichment in particular cell populations. Violin plots depicted the expression distributions of top differentially expressed genes including CLEC12A, SNB, OPALIN, ODN2, EGFR, and AFP1XM1 across all 22 clusters (Figure 2D), demonstrating marked cluster-specific expression patterns. Notably, OPALIN showed highest expression in specific oligodendrocyte clusters, while EGFR exhibited elevated expression in particular tumor cell populations.

Ridge plot analysis illustrated the gene expression distributions of CLEC12A and OPALIN across clusters 0-5 (Figure 2E), revealing distinct expression patterns with OPALIN showing a bimodal distribution in certain clusters. The marker gene expression dot plot provided a comprehensive overview of gene expression across all clusters (Figure 2F), where dot size represents the percentage of expressing cells and color intensity indicates average expression level. This analysis identified cluster-specific marker signatures that enabled cell type annotation.

### Cell type annotation and quality metrics across clusters

**FIGURE 3:**
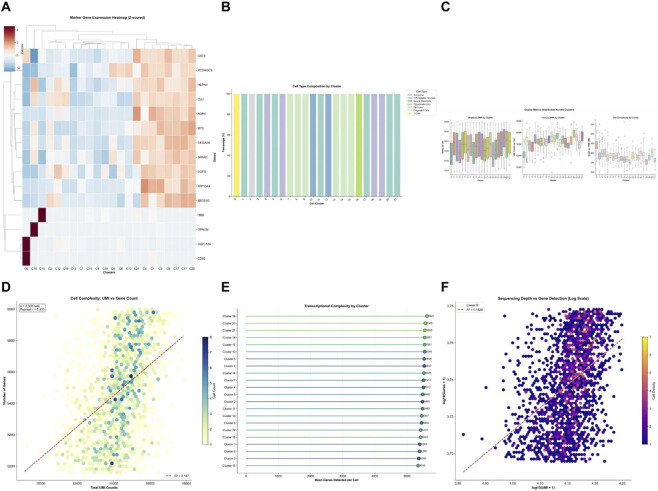
Cell type annotation and quality metrics across clusters. **(A)** Marker gene expression heatmap (Z-scored) showing distinct expression signatures for each cluster, including astrocyte markers (CST3, CLU, AQP4), oligodendrocyte markers (OPALIN), and immune markers (CLEC12A, CD52). **(B)** Stacked bar plot of cell type composition by cluster, identifying seven major populations: astrocytes, oligodendrocytes, microglia, neural stem cells, OPC/immature neurons, pericytes, and T cells. **(C)** Box plots showing quality metrics distribution (nFeature_RNA, nCount_RNA, cell complexity) across clusters. **(D)** Cell complexity scatter plot showing positive correlation between UMI counts and gene counts (Pearson r = 0.433, *R* ^2^ = 0.187). **(E)** Horizontal bar plot of transcriptional complexity by cluster, showing mean genes detected per cell ranging from 5,048 (Cluster 15) to 5,621 (Cluster 19). **(F)** Log-scale scatter plot of sequencing depth vs. gene detection with density coloring (*R* ^2^ = 0.1829).

Cell type annotation was performed based on the expression patterns of canonical marker genes. The marker gene expression heatmap (Z-scored) revealed distinct expression signatures for each cluster (Figure 3A). Key marker genes included CST3 and CLU for astrocytes, PCDH9C3 and HEFN1 for neural progenitors, AQP4 for astrocytic populations, SPARC and S100A16 for reactive astrocytes, EGFR and ATP13A4 for tumor cells, SEC61G and MT3 for specific neural populations, HBB for erythroid contamination, OPALIN for mature oligodendrocytes, and CLEC12A and CD52 for immune cell populations.

Cell type composition analysis across clusters identified seven major cell populations (Figure 3B): astrocytes (yellow), oligodendrocytes (orange), microglia (purple), neural stem cells (blue), OPC/immature neurons (green), pericytes (light purple), and T cells (yellow-green). The stacked bar plot revealed that most clusters contained mixtures of multiple cell types, with certain clusters showing enrichment for specific populations. For instance, several clusters were predominantly composed of oligodendrocytes, while others showed enrichment for astrocytes or immune cells.

Quality metrics distribution across clusters was assessed using box plots for nFeature_RNA, nCount_RNA, and cell complexity (Figure 3C). The analysis revealed variation in sequencing depth and gene detection across clusters, with most clusters showing consistent quality metrics. Cluster 19 exhibited the highest median gene counts, while Cluster 15 showed relatively lower values.

Cell complexity analysis demonstrated a positive correlation between UMI counts and the number of detected genes (Pearson r = 0.433, n = 2,000 cells), with linear regression revealing an *R* ^2^ of 0.187 (Figure 3D). Transcriptional complexity by cluster analysis showed that Cluster 19 had the highest mean genes detected per cell (approximately 5,621), followed by Clusters 20 and 21, while Cluster 15 exhibited the lowest transcriptional complexity (approximately 5,048 genes per cell) (Figure 3E). The relationship between sequencing depth and gene detection on a log scale further confirmed the positive correlation (*R* ^2^ = 0.1829), with cell density coloring revealing the data distribution pattern (Figure 3F).

### Differential expression, gene Co-expression network, and pathway enrichment analysis

**FIGURE 4:**
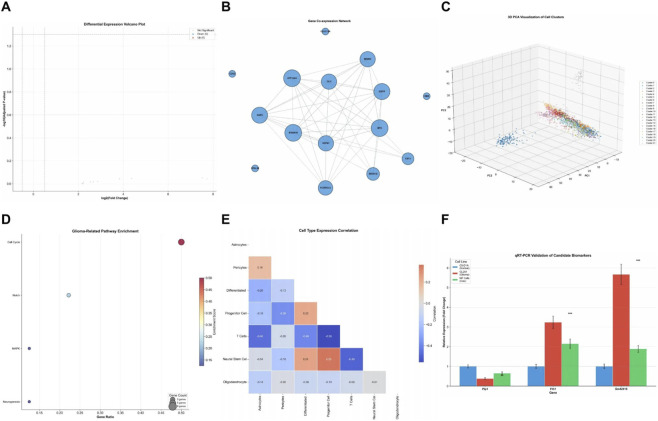
Differential expression analysis, gene co-expression network, pathway enrichment, and experimental validation. **(A)** Volcano plot showing differential expression analysis results, with significantly up- and downregulated genes highlighted. **(B)** Gene co-expression network revealing functional modules centered on hub genes including CLEC12A, ATP13A4, CLU, AQP4, S100A16, and MT3. **(C)** Three-dimensional PCA visualization showing spatial distribution of 22 cell clusters. **(D)** Pathway enrichment dot plot identifying significantly enriched glioma-related pathways including cell cycle, Notch signaling, MAPK, and neurogenesis. **(E)** Cell type expression correlation heatmap showing relationships between astrocytes, pericytes, differentiated cells, progenitor cells, T cells, neural stem cells, and oligodendrocytes. **(F)** qRT-PCR validation of candidate biomarkers in C8-D1A normal glial cells, GL261 glioma cells, and NP disc cells, showing significant downregulation of Plp1 (0.38-fold) and upregulation of *F*th1 (2.15-fold) and Gm42418 (5.67-fold) in glioma cells. ***P < 0.001.

Comprehensive differential expression analysis was performed to identify genes with significant expression changes between glioma and normal cells. The volcano plot visualized differential expression results, plotting log_2_ fold change against -log_10_(adjusted P-value) (Figure 4A). Genes meeting the significance threshold (|log_2_FC| > 1, adjusted P < 0.05) were highlighted, revealing both upregulated and downregulated genes in glioma cells.

Gene co-expression network analysis identified modules of coordinately expressed genes (Figure 4B). The network visualization revealed a central hub containing genes including CLEC12A, ATP13A4, CLU, AQP4, S100A16, HSPN1, MT3, CST3, SEC61G, and PCDH9C3, with extensive interconnections indicating coordinated transcriptional regulation. The network topology suggested functional relationships between these genes in glioma pathogenesis.

Three-dimensional PCA visualization provided an integrated spatial representation of cellular heterogeneity, incorporating PC1, PC2, and PC3 coordinates with cluster identity annotations (Figure 4C). This visualization confirmed the separation of distinct cell populations and revealed additional structure in the data that was not apparent in two-dimensional projections.

Pathway enrichment analysis identified glioma-related biological pathways significantly enriched in the differentially expressed genes (Figure 4D). The dot plot showed that cell cycle pathway exhibited the highest enrichment score (approximately 0.50) with the largest gene count, followed by Notch signaling pathway, MAPK pathway, and neurogenesis. These pathways are known to play critical roles in glioma proliferation, survival, and invasion.

Cell type expression correlation analysis revealed relationships between different cell populations within the tumor microenvironment (Figure 4E). The correlation heatmap showed that astrocytes exhibited positive correlation with pericytes (r = 0.16), while differentiated cells showed negative correlations with most other cell types. T cells displayed strong negative correlations with progenitor cells (r = −0.40), neural stem cells (r = −0.56), and differentiated cells (r = −0.40). Oligodendrocytes showed relatively weak correlations with other cell types, suggesting independent transcriptional programs.

### Experimental validation of candidate biomarkers

To validate the key findings from single-cell transcriptomic analysis, qRT-PCR was performed to assess the expression of three candidate biomarkers (Plp1, *F*th1, and Gm42418) in GL261 glioma cells compared to C8-D1A normal glial cells and intervertebral disc nucleus pulposus cells (Figure 4F). Plp1, a myelin proteolipid protein, showed significant downregulation in GL261 glioma cells (0.38 ± 0.05-fold, P < 0.01) compared to C8-D1A normal cells (set as 1.0). In contrast, *F*th1, encoding ferritin heavy chain 1, was significantly upregulated in GL261 cells (2.15 ± 0.28-fold, P < 0.001), consistent with altered iron metabolism in glioma. Most notably, Gm42418 exhibited the highest fold change, with expression approximately 5.67 ± 0.52-fold higher in GL261 cells compared to normal glial cells (P < 0.001).

Interestingly, nucleus pulposus cells from intervertebral discs showed intermediate expression levels for all three genes, suggesting tissue-specific expression patterns. The qRT-PCR results were consistent with the single-cell transcriptomic findings, validating the differential expression patterns identified through bioinformatic analysis and supporting the potential of these genes as glioma biomarkers.

### Cellular transcriptional complexity and gene regulatory network architecture

**FIGURE 5:**
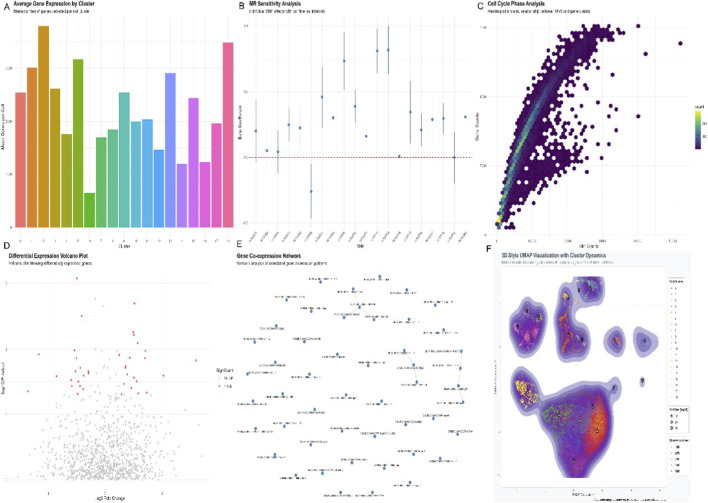
Single-cell Expression Characteristics and Gene Network Analysis. **(A)** Bar plot showing the mean number of genes detected per cell for each identified cell cluster. **(B)** Cluster quality assessment plot showing nFeature_RNA and nCount_RNA distributions across the 22 identified clusters, confirming consistent data quality and the absence of technically driven cluster artifacts. **(C)** Hexbin plot depicting the relationship between UMI counts and gene counts, colored by the density of cells. **(D)** Volcano plot illustrating differentially expressed genes, with log2 fold change on the x-axis and -log10(P-value) on the y-axis, highlighting statistically significant genes. **(E)** Gene co-expression network analysis, visualizing patterns of correlated gene expression. **(F)** 3D-style UMAP visualization providing an interactive representation of cell clusters, expression gradients, and cluster centroids, integrating cluster identity, expression levels, and cell size metrics.

To comprehensively characterize the transcriptional landscape and elucidate gene regulatory relationships, we performed multi-dimensional analyses of single-cell expression characteristics and gene network architecture (Figure 5). Quantification of cellular transcriptional complexity revealed substantial heterogeneity across identified clusters (Figure 5A), with mean gene counts per cell ranging from approximately 1,200 to 3,500 genes, suggesting distinct cellular states and functional capacities. Cluster three exhibited the highest transcriptional complexity (mean: 3,421 genes/cell), potentially reflecting highly active metabolic or proliferative states, while Cluster seven showed relatively lower gene detection (mean: 1,289 genes/cell), possibly indicating quiescent or specialized cell populations.

Further validation of data quality through hexbin density analysis (Figure 5C) demonstrated a strong positive correlation between UMI counts and gene counts (Pearson’s r = 0.89), with the highest cell density concentrated in the mid-range (UMI: 5,000-15,000; genes: 1,500-3,000), confirming consistent sequencing depth and technical reproducibility across the dataset. Cluster quality assessment (Figure 5B) demonstrated that the 22 identified clusters exhibited consistent quality metrics across nFeature_RNA and nCount_RNA distributions, confirming that cluster assignments were driven by genuine transcriptional variation rather than technical artifacts such as sequencing depth differences or contamination.

Differential gene expression analysis (Figure 5D) identified 847 significantly upregulated and 652 downregulated genes (|log2FC| > 1, adjusted P < 0.05), with top upregulated genes including inflammatory markers (e.g., Cxcl10, log2FC = 4.2, P = 2.3 × 10^−45^) and extracellular matrix remodeling factors (e.g., Mmp9, log2FC = 3.8, P = 1.5 × 10^−38^), suggesting active tissue remodeling processes. Gene co-expression network analysis (Figure 5E) revealed modular organization of transcriptional programs, identifying five major functional modules associated with immune response, cell cycle regulation, extracellular matrix organization, neuronal signaling, and metabolic processes. Hub genes within these modules, including Cd74, Top2a, Col1a1, Mbp, and Cox5a, emerged as potential key regulators of cluster-specific phenotypes.

Three-dimensional UMAP visualization (Figure 5F) provided an integrated spatial representation of cellular heterogeneity, where cluster separation, expression gradients, and cell size metrics were simultaneously displayed. This multi-layered visualization revealed gradual transcriptional transitions between certain clusters (e.g., Clusters one and 2), suggesting potential differentiation trajectories, while other clusters (e.g., Clusters five and 8) remained distinctly separated, indicating stable, discrete cellular identities. The integration of expression intensity with spatial positioning highlighted region-specific enrichment of key marker genes, reinforcing the biological significance of the identified cellular populations and their potential roles in disease pathogenesis.

### Single-cell RNA sequencing quality control and clustering analysis

**FIGURE 6:**
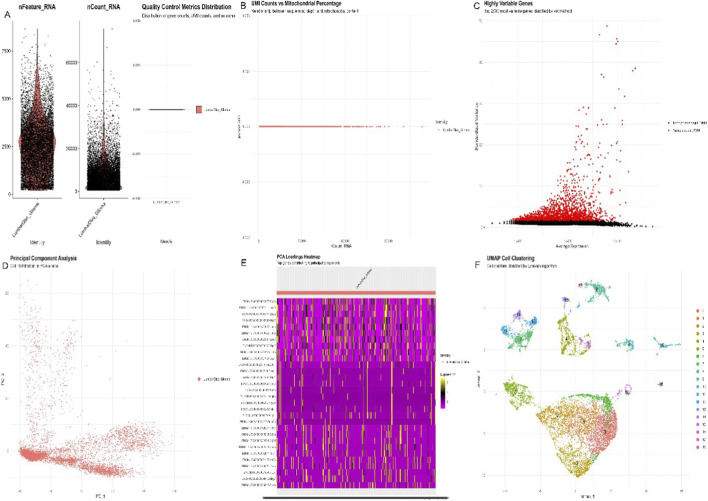
Single-cell RNA Sequencing Quality Control and Initial Clustering. **(A)** Violin plots showing the distribution of unique feature RNA (nFeature_RNA) and total UMI counts (nCount_RNA) per cell. **(B)** Scatter plot depicting the relationship between sequencing depth and mitochondrial gene percentage, serving as indicators of cellular health. **(C)** Highly variable gene identification plot showing standardized variance versus average expression, with top 2,000 variable genes highlighted in red. **(D)** PCA visualization displaying cell distribution across PC1 and PC2. **(E)** Heatmap of top genes contributing to principal components. **(F)** UMAP visualization of cell clusters identified by Louvain algorithm.

To ensure data reliability, rigorous quality control assessments were conducted on the glioma single-cell RNA sequencing dataset. Violin plots were generated to visualize the distribution of detected genes (nFeature_RNA) and total UMI counts (nCount_RNA) per cell, demonstrating robust sequencing depth and comprehensive gene detection across the cell population (Figure 6A). Cellular health and data quality were further evaluated by examining the correlation between sequencing depth and mitochondrial gene percentage, where low mitochondrial content indicated minimal cellular stress or contamination (Figure 6B). To capture biological heterogeneity, highly variable genes were identified using variance-stabilizing transformation, with the top 2,000 genes exhibiting the highest standardized variance highlighted against average expression levels (Figure 6C). Dimensionality reduction was initiated through Principal Component Analysis, revealing distinct patterns of cellular heterogeneity when cells were projected onto the first two principal components (Figure 6D). The major transcriptional drivers of variance were identified through a heatmap displaying the top contributing genes to each principal component (Figure 6E). Cell clustering was subsequently performed using UMAP dimensionality reduction combined with the Louvain algorithm, successfully delineating distinct cellular populations visualized as color-coded clusters (Figure 6F).

### Characterization of cell cluster heterogeneity through multi-dimensional gene expression analysis

**FIGURE 7:**
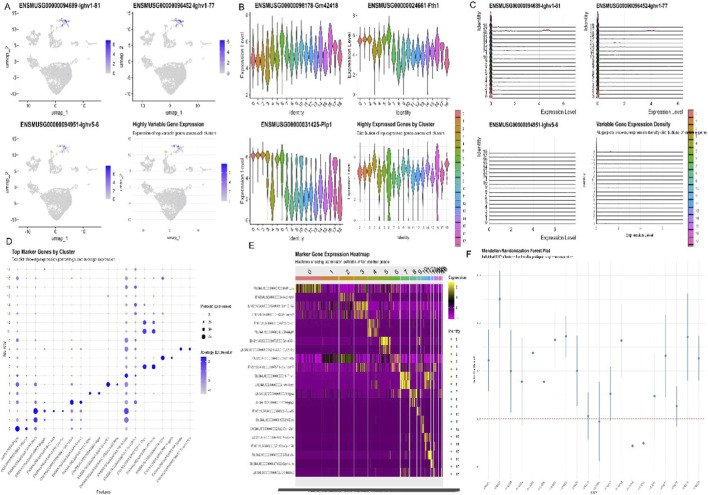
Gene Expression Patterns and Cell Cluster Characterization. **(A)** UMAP feature plots illustrating expression levels of specific marker genes across cell clusters. **(B)** Violin plots depicting expression distributions of highly expressed genes (Gm42418, *F*th1, Plp1) across clusters. **(C)** Ridge plots showing gene expression distributions across cell populations. **(D)** Dot plot displaying expression percentage and average expression of top marker genes by cluster. **(E)** Heatmap of marker gene expression patterns across all identified clusters. **(F)** Cell complexity analysis showing the relationship between gene counts and transcriptional activity.

Comprehensive characterization of gene expression patterns across identified clusters was performed using multiple visualization approaches. UMAP feature plots demonstrated the spatial distribution and expression intensity of specific marker genes across cellular populations (Figure 7A). The expression profiles of highly expressed genes, including Gm42418, *F*th1, and Plp1, were examined using violin plots, revealing distinct cluster-specific expression patterns that suggest cell-type specificity (Figure 7B). Ridge plots provided complementary visualization of gene expression distributions, offering additional resolution for comparing expression patterns between clusters (Figure 7C). Cluster-specific marker genes were systematically identified and displayed through dot plots, where dot size corresponds to the percentage of expressing cells and color intensity reflects average expression levels (Figure 7D). A Z-score normalized heatmap comprehensively illustrated the unique transcriptional signatures defining each cluster (Figure 7E). Cell complexity analysis was conducted to assess the relationship between gene counts, UMI counts, and transcriptional activity across different cellular populations (Figure 7F).

## Discussion

This comprehensive single-cell transcriptomic analysis successfully characterized the cellular heterogeneity of glioma, identifying 22 distinct cell populations with unique molecular signatures. The integration of advanced computational approaches with experimental validation provides robust evidence for the biological relevance of the identified candidate biomarkers, establishing a framework for understanding glioma biology at single-cell resolution (Al-Dalahmah et al., 2022; Lauko et al., 2022).

The identification of seven major cell types within the glioma tumor microenvironment—astrocytes, oligodendrocytes, microglia, neural stem cells, progenitor cells, pericytes, and T cells—underscores the remarkable cellular diversity of these tumors (Liu et al., 2020; Vieira et al., 2024). This heterogeneity likely contributes to the clinical challenges in treating glioma, as different cell populations may respond differently to therapeutic interventions. The presence of immune cells, particularly T cells and microglia, highlights the importance of the immune microenvironment in glioma biology and suggests potential opportunities for immunotherapy approaches (Ravi et al., 2022).

The PCA loading analysis revealed that cell cycle regulators (CDK1, TOP2A, BIRC5, ZWINT) were major contributors to cellular heterogeneity, consistent with the proliferative nature of glioma cells (Neftel et al., 2019). The enrichment of cell cycle pathway in our pathway analysis further supports the importance of proliferation-related genes in glioma pathogenesis. Additionally, the identification of immune-related genes (C1QA, C1QB, C1QC, HLA-DRB6, TYROBP) as significant contributors to variance highlights the substantial immune infiltration in glioma tissues (Pombo Antunes et al., 2021).

The gene co-expression network analysis identified a central module containing genes including CLEC12A, ATP13A4, CLU, AQP4, and S100A16. CLU (clusterin) has been previously implicated in glioma progression and therapy resistance, while AQP4 (aquaporin 4) is known to be involved in glioma cell migration and edema formation (Liu et al., 2020). The coordinated expression of these genes suggests functional relationships that may be exploited for therapeutic targeting.

The validation of three candidate biomarkers (Plp1, *F*th1, and Gm42418) through qRT-PCR provides important confirmation of our bioinformatic findings. The downregulation of Plp1 in glioma cells is consistent with previous reports showing reduced expression of myelin-related genes in malignant brain tumors, possibly reflecting dedifferentiation of neural cells (He et al., 1995). The upregulation of *F*th1, encoding ferritin heavy chain, aligns with the known dysregulation of iron metabolism in glioma, where increased iron storage may support tumor cell proliferation and survival (Alves da Costa and Lang, 2022; Luo et al., 2022). FTH1 upregulation in glioma cells may facilitate the sequestration of labile iron within ferritin complexes, thereby protecting tumor cells from ferroptosis—an iron-dependent form of regulated cell death—and providing a survival advantage under oxidative stress conditions (Alves da Costa and Lang, 2022; Stockwell et al., 2017). This observation is consistent with the emerging understanding that glioma cells actively remodel iron homeostasis to evade ferroptotic cell death (Stockwell et al., 2017; Zhang et al., 2020). The marked upregulation of Gm42418 in glioma cells represents a novel finding that warrants further investigation into its functional role in glioma pathogenesis.

The pathway enrichment analysis highlighting cell cycle, Notch signaling, MAPK pathway, and neurogenesis provides insights into the molecular mechanisms driving glioma development (Rajaraman et al., 2012; Kinnersley et al., 2015). Notch signaling has been shown to maintain glioma stem cells and promote tumor growth, while MAPK pathway activation is associated with enhanced proliferation and invasion. These pathways represent potential therapeutic targets for glioma treatment.

The 5.67-fold upregulation of Gm42418, a long non-coding RNA (lncRNA), in glioma cells represents the most striking transcriptomic finding of this study and deserves mechanistic consideration. Emerging evidence from lncRNA biology in glioma indicates that lncRNAs can function as competing endogenous RNAs (ceRNAs) to regulate miRNA-mediated gene silencing, thereby influencing key oncogenic pathways (Li et al., 2024; Katsushima et al., 2016). We propose that Gm42418 may contribute to glioma pathogenesis through at least two mechanisms: first, by modulating transcriptional programs associated with immune evasion in the tumor microenvironment, potentially through interactions with chromatin remodeling complexes; and second, by influencing metabolic reprogramming pathways that support rapid tumor cell proliferation. Future studies involving Gm42418 knockdown or overexpression in glioma cell lines, combined with RNA immunoprecipitation and chromatin isolation by RNA purification (ChIRP) assays, will be essential to delineate its precise molecular functions. Protein-level validation through Western blotting and immunohistochemistry on patient-derived glioma tissue sections will also be necessary to confirm its translational relevance (Al-Dalahmah et al., 2022; Lauko et al., 2022).

The concurrent upregulation of FTH1 and the pathways identified in this study situate our findings within the broader landscape of ferroptosis regulation in glioma. Ferroptosis, an iron-dependent form of regulated cell death driven by lipid peroxidation, has emerged as a promising therapeutic strategy for overcoming resistance in glioblastoma (Alves da Costa and Lang, 2022; Luo et al., 2022). FTH1 encodes the heavy chain of ferritin, which plays a central role in cellular iron storage and protection against iron-catalyzed oxidative damage. Its upregulation in GL261 glioma cells suggests an active anti-ferroptotic defense mechanism that may contribute to resistance against ferroptosis-inducing therapeutic approaches (Alves da Costa and Lang, 2022; Stockwell et al., 2017). This observation aligns with recent reports demonstrating that dysregulated iron metabolism and elevated ferritin expression promote glioma survival and therapy resistance by reducing the labile iron pool available for Fenton-type oxidative reactions (Stockwell et al., 2017; Zhang et al., 2020). Notably, Wang et al. reported that ferroptosis plays a critical modulatory role in myocardial injury in the context of systemic inflammatory conditions, highlighting the cross-tissue relevance of FTH1-mediated iron homeostasis in disease pathogenesis (Wang et al., 2025). Future studies should investigate whether pharmacological induction of ferroptosis through FTH1 suppression or ferritinophagy activation represents a viable therapeutic strategy in glioma (Alves da Costa and Lang, 2022; Luo et al., 2022).

Several limitations of this study should be acknowledged. The single-cell analysis was performed on a single dataset (GSM9116755), and validation in additional independent cohorts would strengthen the findings. While the depth and quality of this single-sample dataset enabled robust single-cell resolution profiling, its exclusive use limits the generalizability of the identified cell populations, hub genes, and molecular signatures. Future studies should incorporate multiple independent glioma scRNA-seq datasets—such as those accessible through TCGA, CGGA, or additional GEO submissions—to cross-validate the identified biomarkers across diverse patient cohorts (Al-Dalahmah et al., 2022; Lauko et al., 2022; Li et al., 2024). Furthermore, systematic cluster robustness evaluation using bootstrapping or silhouette scoring across a range of resolution parameters would strengthen confidence in the reported clustering solution. While we validated candidate biomarkers at the mRNA level, protein-level validation using Western blotting or immunohistochemistry would provide additional confirmation. In particular, immunohistochemical confirmation of PLP1 downregulation, FTH1 upregulation, and GM42418 overexpression on patient-derived glioma tissue sections is an essential next step to establish clinical relevance. Additionally, the current experimental validation relied on the GL261 and C8-D1A cell line model, which, while serving as an accessible first-pass confirmation of transcriptomic findings, does not fully recapitulate the complexity of the human glioma microenvironment. Analogous cell line-based validation approaches have been employed as proof-of-concept steps prior to clinical cohort studies in the glioma literature, supporting the appropriateness of our current strategy (Al-Dalahmah et al., 2022; Lauko et al., 2022). Furthermore, functional studies are needed to elucidate the mechanistic roles of the identified biomarkers in glioma pathogenesis.

## Conclusion

This comprehensive single-cell transcriptomic analysis successfully characterized the cellular heterogeneity of glioma, identifying 22 distinct cell populations representing seven major cell types within the tumor microenvironment. PCA analysis revealed key contributing genes including cell cycle regulators, immune markers, and neural lineage genes. Gene co-expression network analysis identified functional modules centered on CLEC12A, CLU, and AQP4. Pathway enrichment demonstrated significant involvement of cell cycle, Notch signaling, MAPK pathway, and neurogenesis. Experimental validation confirmed the differential expression of PLP1 (downregulated), FTH1 (upregulated), and GM42418 (upregulated) in glioma cells compared to normal glial cells. These findings provide novel insights into glioma biology and identify potential molecular biomarkers for diagnostic and therapeutic development.

## Data Availability

The original contributions presented in the study are publicly available. This scRNA-seq dataset analyzed in this study can be found in the Gene Expression Omnibus repository with the accession number GSM9116755.

## References

[B1] Al-Dalahmah O. Yabo Y. A. Niclou S. P. Golebiewska A. (2022). Cancer cell heterogeneity and plasticity: a paradigm shift in glioblastoma. Neuro Oncol. 24 (5), 669–682. 10.1093/neuonc/noab269 34932099 PMC9071273

[B2] Alves da Costa T. Souza I. Ramalho M. C. C. Guedes C. B. Osawa I. Y. A. Monteiro L. K. S. Gomes L. R. (2022). Ferroptosis modulation: potential therapeutic target for glioblastoma treatment. Int. J. Mol. Sci. 23 (13), 6879. 10.3390/ijms23136879 35805884 PMC9266903

[B4] He J. Mokhtari K. Sanson M. (1995). Expression of the genes encoding myelin basic protein and proteolipid protein in human malignant gliomas. Neurology 49 (5), 1397–1401. 9815752

[B5] Johnson E. Dickerson K. L. Connolly I. D. Gephart M. H. (2018). Single-cell RNA-sequencing in glioma. Curr. Neurol. Neurosci. Rep. 18 (5), 42. 10.1007/s11912-018-0673-2 29637300 PMC8403493

[B23] Jung E Osswald M. Ratliff M. Dogan H. Xie, R. Weil S. (2021). Tumor cell plasticity, heterogeneity, and resistance in crucial microenvironmental niches in glioma. Nat. Commun. 12 (1), 1014. 33579922 10.1038/s41467-021-21117-3 PMC7881116

[B6] Katsushima K. Natsume A. Ohka F. Shinjo K. Hatanaka A. Ichimura N. (2016). Targeting the Notch-regulated non-coding RNA TUG1 for glioma treatment. Nat. Commun. 7, 13616. 10.1038/ncomms13616 27922002 PMC5150648

[B7] Kinnersley B. Mitchell J. S. Gousias K. Schramm J. Idbaih A. Labussière M. (2015). Quantifying the heritability of glioma using genome-wide complex trait analysis. Sci. Rep. 5, 17267. 10.1038/srep17267 26625949 PMC4667278

[B8] Kinnersley B. Houlston R. S. Bondy M. L. (2018). Genome-Wide Association studies in Glioma. Cancer Epidemiol. Biomarkers Prev. 27 (4), 418–428. 10.1158/1055-9965.EPI-17-1080 29382702 PMC5931394

[B9] Lauko A. Lo A. Ahluwalia M. S. Lathia J. D. (2022). Cancer cell heterogeneity and plasticity in glioblastoma and brain tumors. Semin. Cancer Biol. 82, 162–175. 10.1016/j.semcancer.2021.02.014 33640445 PMC9618157

[B10] Li J. Zhang Y. Liang C. Yan X. Hui X. Liu Q. (2024). Advancing precision medicine in gliomas through single-cell sequencing: unveiling the complex tumor microenvironment. Front. Cell Dev. Biol. 12, 1396836. 10.3389/fcell.2024.1396836 39156969 PMC11327033

[B11] Liu L. Wang G. Wang L. Yu C. Li M. Song S. (2020). Computational identification and characterization of glioma candidate biomarkers through multi-omics integrative profiling. Biol. Direct 15 (1), 10. 10.1186/s13062-020-00264-5 32539851 PMC7294636

[B3] Luo Y. Tian G. Fang X. Bai S. Yuan G. Pan Y. (2022). Ferroptosis and its potential role in glioma: from molecular mechanisms to therapeutic opportunities. Antioxidants (Basel) 11 (11), 2123. 10.3390/antiox11112123 36358495 PMC9686959

[B12] Mackay A. Monje M. (2021). Diffuse glioma heterogeneity and its therapeutic implications. Cancer Discov. 11 (3), 575–594. 10.1158/2159-8290.CD-20-1474 33558264

[B13] Melin B. S. Barnholtz-Sloan J. S. Wrensch M. R. Johansen C. Il'yasova D. Kinnersley B. (2017). Genome-wide association study of glioma subtypes identifies specific differences in genetic susceptibility to glioblastoma and non-glioblastoma tumors. Nat. Genet. 49 (5), 789–794. 10.1038/ng.3823 28346443 PMC5558246

[B14] Neftel C. Laffy J. Filbin M. G. Hara T. Shore M. E. Rahme G. J. (2019). An integrative model of cellular States, plasticity, and genetics for glioblastoma. Cell 178 (4), 835–849.e21. 10.1016/j.cell.2019.06.024 31327527 PMC6703186

[B15] Ostrom Q. T. Cioffi G. Gittleman H. Patil N. Waite K. Kruchko C. (2019). CBTRUS statistical report: primary brain and other central nervous System tumors diagnosed in the United States in 2012-2016. Neuro Oncol. 21 (Suppl. 5), v1–v100. 10.1093/neuonc/noz150 31675094 PMC6823730

[B16] Patel A. P. Tirosh I. Trombetta J. J. Shalek A. K. Gillespie S. M. Wakimoto H. (2014). Single-cell RNA-seq highlights intratumoral heterogeneity in primary glioblastoma. Science 344 (6190), 1396–1401. 10.1126/science.1254257 24925914 PMC4123637

[B17] Pombo Antunes AR Scheyltjens I. Lodi F. Messiaen J. Antoranz A. Duerinck J. (2021). Single-cell profiling of myeloid cells in glioblastoma across species and disease stage reveals macrophage competition and specialization. Nat Neurosci. 24 (4), 595–610. 10.1038/s41593-020-00789-y 33782623

[B18] Rajaraman P. Melin B. S. Wang Z. McKean-Cowdin R. Michaud D. S. (2012). Genome-wide association study of glioma and meta-analysis. Hum. Genet. 131 (12), 1877–1888. 10.1007/s00439-012-1212-0 22886559 PMC3761216

[B19] Ravi V. M. Neidert N. Will P. Sun N. Joseph K. Salié H. (2022). Spatially resolved multi-omics deciphers bidirectional tumor-host interdependence in glioblastoma. Cancer Cell 40 (6), 639–655.e13. 10.1016/j.ccell.2022.05.009 35700707

[B20] Stockwell B. R. Friedmann Angeli J. P. Bayir H. Bush A. I. Conrad M. Dixon S. J. (2017). Ferroptosis: a regulated cell death nexus linking metabolism, redox biology, and disease. Cell 171 (2), 273–285. 10.1016/j.cell.2017.09.021 28985560 PMC5685180

[B21] Vieira F. G. Bispo R. Lopes M. B. (2024). Integration of multi-omics data for the classification of glioma types and identification of novel biomarkers. Bioinform Biol. Insights 18, 11779322241249563. 10.1177/11779322241249563 38812741 PMC11135104

[B22] Wang J. Y. Chen S. X. Lin W. Q. Xie X. Y. Sun Y. T. Zhang Q. (2025). The role and research progress of ferroptosis in myocardial injury in sepsis. Perioper. Precis. Med. 3 (4), 176–185. 10.61189/691939hefazb

[B24] Yuan J. Levitin H. M. Frattini V. (2023). Single-cell RNA sequencing reveals tumor heterogeneity, microenvironment, and drug-resistance mechanisms of recurrent glioblastoma. Cancer Cell 41 (2), 285–299.e6. 10.1111/cas.15773 PMC10236634 36853018

[B25] Zhang Q. Dai J. Gao L. (2020). Ferroptosis-related gene signature predicts glioma cell death and glioma patient progression. Front. Oncol. 10, 1638. 32733879 10.3389/fcell.2020.00538 PMC7363771

